# A novel bispecific nanobody protects mice against RSV infection via intranasal administration

**DOI:** 10.1128/jvi.01285-25

**Published:** 2025-11-24

**Authors:** Min Zhang, Liuxing Qin, Raoqing Guo, Jinwei Li, Si Huang, Chen Sheng, Yijia Xiang

**Affiliations:** 1 State Key Laboratory of Respiratory Disease, National Clinical Research Center for Respiratory Disease, Guangzhou Institute of Respiratory Health, The First Affiliated Hospital of Guangzhou Medical University, Guangzhou Medical University 26468, Guangzhou, Guangdong, China; 2 Guangzhou National Laboratory, Bio-island 26468, Guangzhou, Guangdong, China; 3 Guangzhou Medical University, Guangzhou, Guangdong, China; St Jude Children's Research Hospital, Memphis, Tennessee, USA

**Keywords:** respiratory syncytial virus, bispecific nanobodies, albumin-binding, intranasal administration, antigenic site Ø

## Abstract

Respiratory syncytial virus (RSV) is the leading cause of respiratory infection-related hospitalizations in children younger than 5 years. Neutralizing nanobody-based interventions represent a promising strategy against RSV. Here, we identify a novel nanobody (4-H1) targeting the RSV prefusion F (pre-F) protein, which demonstrates potent neutralization against both RSV A and B subtypes. Epitope characterization via binning assays, molecular docking, and mutational analyses revealed that 4-H1 interacts with a unique region within antigenic site Ø by engaging critical residues L207, K209, and the K65-N67-C69 cluster. To improve the *in vivo* efficacy and stability of the 4-H1 nanobody, we engineered a heterotrimeric bispecific nanobody (4-H1-anti-HSA-4-H1). This single-chain molecule contains two anti-RSV F nanobody domains and one anti-human serum albumin (HSA) domain, resulting in a trivalent molecule with dual specificity. This construct demonstrated sub-nanogram per milliliter (sub-ng/mL) neutralization potency against both RSV A and B subtypes, with prolonged *in vivo* half-life. Notably, intranasal administration of this construct before exposure conferred robust protection against RSV challenge in BALB/c mice. These results underscore the potential of 4-H1-anti-HSA-4-H1 as a respiratory-delivered prophylactic against RSV.

**IMPORTANCE:**

RSV is the leading cause of infant respiratory hospitalizations, highlighting the urgent need for effective prophylaxis. Here, we engineered a potent bispecific nanobody (4-H1-anti-HSA-4-H1) that exhibits exceptional neutralization against both RSV A and B subtypes with prolonged serum persistence. Prophylactic intranasal delivery of this construct conferred robust protection against RSV challenge in mice, indicating its potential as a respiratory-delivered prophylactic candidate against RSV.

## INTRODUCTION

RSV is the predominant cause of severe lower respiratory tract infections, including bronchiolitis and pneumonia, in infants and young children (1). Globally, in 2019, RSV caused 33.0 million episodes of acute lower respiratory infections (ALRI) in children younger than 5 years of age, resulting in 3.6 million hospital admissions and 101,400 deaths (2, 3). Currently, no vaccine is approved for the active immunization of infants against RSV. Instead, protection in this population is achieved through passive immunization strategies, which include one maternal vaccine that provides passive immunity to infants (4), as well as three prophylactic monoclonal antibodies (mAbs). Palivizumab was the first mAb approved for RSV prevention. However, its use requires multiple doses and is approved only for high-risk infants (5). More recently developed mAbs, including nirsevimab and clesrovimab, offer single-dose prophylaxis and have substantially expanded patient eligibility (6, 7). Despite these advancements, persistent challenges related to cost, equitable access, and real-world implementation underscore the ongoing need for novel interventions.

RSV strains comprise two antigenic subtypes (A and B) and possess a negative-sense single-stranded RNA genome encoding 11 viral proteins. Among them, the surface fusion glycoprotein (F) is highly conserved across RSV isolates from both subgroups, making it a prime target for vaccine and drug development (8, 9). This metastable protein exists in two conformational states: a prefusion form (pre-F) that transitions either spontaneously or upon cell attachment to the stable postfusion form (post-F). The pre-F conformation displays six major antigenic sites (Ø, I–V), whereas only sites I–IV persist in the post-F form (10, 11). Antibodies targeting pre-F-specific sites Ø or V demonstrate superior neutralizing potency, exemplified by nirsevimab (site Ø-specific), which exhibits >100-fold higher *in vitro* neutralization potency than palivizumab, a site II-targeting agent, against RSV A2 and RSV B9320 (12). This structural superiority underlies the superior clinical efficacy of nirsevimab (89.6% protection in infants ≤ 3 months), whereas palivizumab is only recommended for high-risk subgroups, including preterm neonates and patients with congenital heart disease or other severe comorbidities (6, 13). Moreover, all three clinically approved RSV vaccines leverage pre-F antigens to elicit potent neutralizing antibodies (4, 14, 15).

In addition to standard immunoglobulins, camelids express heavy-chain-only antibodies (HCAbs) featuring single variable domains (VHHs, also called nanobodies) (16, 17). Nanobodies exhibit distinctive structural simplicity that confers advantages such as efficient tissue penetration, superior stability, and exceptional solubility (18). Their single-gene encoding also enables low-cost production in microbial hosts (*E. coli* or yeast), bypassing the complex glycosylation requirements and substantial expenses inherent to mammalian cell expression systems (19). The extended complementarity-determining region (CDR) loops of nanobodies facilitate the formation of a finger-like or convex paratope, allowing them to penetrate cryptic cavities or bind to concave epitopes inaccessible to conventional antibodies (20, 21). Additionally, nanobodies can be readily engineered into multivalent formats, which are devoid of Fc regions and thus reduce the risk of antibody-dependent enhancement (ADE) observed in viral infections such as dengue virus, human immunodeficiency virus (HIV), severe acute respiratory syndrome coronavirus (SARS-CoV), SARS-CoV-2, Middle East respiratory syndrome coronavirus (MERS-CoV), and RSV (22–26). Multivalency further enhances binding avidity and broadens antigen recognition. These biophysical and economic advantages accelerate the development of potent antiviral nanobodies against RSV and related pathogens (27–32).

To date, several RSV F-targeting nanobodies have demonstrated *in vitro* neutralizing activity, with select candidates advancing to *in vivo* evaluation (31, 33–37). Given the superior neutralizing responses elicited by pre-F compared with post-F in murine and non-human primate models (38), we immunized an alpaca with an engineered pre-F-stabilized trimer, DS-Cav1, which has been demonstrated to be a highly immunogenic antigen. Subsequent phage display screening yielded a potent nanobody (4-H1) with neutralizing activity against both RSV subtypes. Building on this scaffold, we engineered the heterotrimeric bispecific construct 4-H1-anti-HSA-4-H1, which simultaneously targets viral pre-F and HSA, achieving extended half-life and effective intranasal protection against RSV *in vivo*. Collectively, these results establish 4-H1-anti-HSA-4-H1 as a promising candidate for respiratory-delivered RSV prophylaxis, warranting further translational development.

## MATERIALS AND METHODS

### Cells and viruses

FreeStyle 293 F cells (Gibco, R79007) were maintained in FreeStyle 293 expression medium (Gibco, 12338018). HEp-2 cells and RSV A2 strain (kindly provided by Dr. Zishu Pan, Wuhan University) were cultured in DMEM (Gibco, C11995500BT) supplemented with 10% FBS (Lonsera, S711-001S), penicillin, and streptomycin (Gibco, 15140122). Viral stocks of RSV strains A2 and B1 were propagated and quantified in HEp-2 cells by plaque assay. Briefly, confluent HEp-2 monolayers in 96-well plates were inoculated with serially diluted RSV for 1 h at 37°C, and then, the cells were overlaid with 1.5% carboxymethylcellulose (Sigma, C4888) in MEM (Gibco, 11900-024) containing 2% FBS. After 72 h of incubation, the cells were fixed and stained with the RB1 mAb specific for RSV F, followed by horseradish peroxidase (HRP)-conjugated goat anti-human IgG (Jackson ImmunoResearch, 109-035-088). Plaques were visualized using TrueBlue Peroxidase Substrate (KPL, 5510-0030) and counted using ImmunoSpot Analyzer (CTL).

### Production and purification of RSV F mAbs

The variable regions of the heavy and light chains from mAbs were synthesized and cloned into a pcDNA3.1 expression vector containing the constant domains of the human IgG1 heavy chain (HC) and κ or λ light chain (LC). To generate the nirsevimab HC variant, YTE mutations (M252Y/S254T/T256E) were introduced into the HC constant region. Equimolar amounts of the HC and LC expression plasmids were transiently transfected into 293 F cells using polyethylenimine (Polysciences, 24765). After 5 days of culture, the cell supernatants were harvested and clarified by centrifugation (10,000 × *g*, 10 min), filtered through a 0.45 µm membrane, and purified with rProtein A Magarose Beads (Smart-Lifesciences, SM003025). The antibodies were dialyzed against PBS and concentrated using 30 kDa Amicon Ultra centrifugal filters (Millipore).

### Production and purification of RSV F proteins

RSV post-F proteins were expressed in 293 F cells via transient transfection of expression plasmids. Pre-F proteins (DS-Cav1 for RSV A2 and B1) were produced using a lentiviral system. The wild-type and mutated DS-Cav1 sequences were synthesized and cloned into the PLVX-EF1α-IRES-Puro lentivirus expression vector (designated lenti-DS-Cav1). Lentiviral particles were generated by co-transfecting 293T cells with lenti-DS-Cav1, VSV-G envelope plasmid pMD2.G, and packaging plasmid psPAX2 using polyethylenimine. Supernatants harvested at 48 h post-transfection were used to transduce fresh 293T cells in the presence of 6 µg/mL polybrene (Beyotime Biotechnology, C0351). After 72 h, the cells were trypsinized and subjected to puromycin selection (1 µg/mL, Sigma, P8833) for 7 days to eliminate non-resistant cells. Puromycin-resistant pools were adapted to serum-free FreeStyle 293 expression medium without puromycin and cultured for 3–4 days. Subsequently, the supernatants were collected, clarified, sterile-filtered, and purified by Ni-NTA affinity chromatography (Cytiva, 17531802). The proteins were dialyzed against PBS, concentrated to 1–2 mg/mL using 30 kDa Amicon Ultra centrifugal filters, flash-frozen in liquid nitrogen, and stored at −80°C until use.

### Isolation of nanobodies

An adult alpaca was immunized subcutaneously with 0.5 mg of DS-Cav1 A2 antigen emulsified in camelid adjuvant (AlpVHHS, 600-000-001). Booster doses (0.25 mg) were administered every 3 weeks. Pre- and post-immunization sera were collected for titer monitoring by ELISA. Briefly, 96-well plates coated with DS-Cav1 A2 and B1 antigens were probed with serially diluted serum. Twenty milliliters of peripheral blood were collected 14 days after the final immunization for peripheral blood mononuclear cells (PBMCs) isolation via Ficoll-Paque density gradient centrifugation. Total RNA was then extracted, reverse-transcribed, and nanobody genes amplified by nested PCR were ligated into SfiI-digested pADL-23c phagemid vectors. These constructs were sequence-verified and electroporated into TG1 electrocompetent cells (Weidi, DE1055). Phage display libraries were generated by superinfection with M13KO7 helper phage (NEB, N0315S), followed by phage precipitation using 20% PEG/2.5 M NaCl. To isolate high-affinity neutralizing nanobodies targeting the Ø and V epitope, competitive biopanning was performed. In rounds 1 and 2, biotinylated DS-Cav1 A2 was immobilized on Dynabeads M-280 Streptavidin beads (Invitrogen, 11205D) and incubated with the phage library. Bound phage particles were eluted with trypsin. In rounds 3 and 4, biotinylated DS-Cav1 was pre-incubated with site I-, II-, III-, and IV-targeting antibodies, followed by incubation with phage particles enriched from the round 2 and 3 eluates. After four rounds of selection, enriched phage clones were screened by phage ELISA. DS-Cav1 was captured on NeutrAvidin-coated plates (Thermo, 31007), and bound phage was detected using an HRP-conjugated anti-M13 antibody (Sino Biological, 11973-MM05T-H). High-affinity clones were expressed in WK6 cells induced with 1 mM IPTG (24 h, 28°C). Cells were pelleted (10,000 × *g*, 10 min, 4°C) post-induction, resuspended in TES buffer (200 mM Tris, 0.5 mM EDTA, 500 mM sucrose, pH 8.0), and lysed at 4°C for 16 h. After centrifugation, the supernatant was purified by Ni-NTA affinity chromatography. Eluted nanobodies were dialyzed against PBS and concentrated using 3 kDa Amicon filters.

### Enzyme-linked immunosorbent assay (ELISA)

ELISAs were performed using 96-well plates coated with 2 µg/mL of respective antigens (RSV A2 and B1 DS-Cav1 or post-F, mutated DS-Cav1, HSA, MSA) overnight at 4°C. After washing with PBST buffer (PBS containing 0.05% Tween-20), plates were blocked with 5% skim milk in PBS for 2 h at room temperature (RT). For serum binding ELISA, blood samples were centrifuged to isolate serum, and serially diluted serum samples were added to the plates and incubated for 2 h at RT. Bound nanobodies were detected using an HRP-conjugated anti-camelid VHH antibody (GenScript, A01861). For antibody binding ELISA, serially diluted test antibodies (motavizumab, 4-H1-Fc, nirsevimab, 5C4, RSD5, anti-HSA, and 4-H1-anti-HSA-4-H1) were added and incubated for 2 h at RT. Detection was performed using HRP-conjugated goat anti-human IgG (Invitrogen, A18817), anti-mouse IgG (Elabscience, E-AB-1001), and anti-camelid VHH antibody, as appropriate. For phage ELISA, phage supernatants were pre-blocked with 20% skim milk in PBS (50 µL skim milk solution mixed with 200 µL phage supernatant) for 1 h at RT before incubation with coated antigens. Bound phages were detected using an HRP-conjugated anti-M13 antibody. All assays were developed using TMB substrate (Biohao, N0160) and stopped with 1 M H₂SO₄; absorbance was measured at 450 nm using a microplate reader. The half-maximal effective concentration (EC₅₀) was calculated by four-parameter logistic regression in GraphPad Prism 9.5.0, defined as the compound concentration producing 50% of the maximal stimulatory effect relative to baseline and maximum response controls.

### Surface plasmon resonance (SPR) binding characterization

SPR binding kinetics were analyzed using a Biacore T200 system. Ligands (anti-HSA, 4-H1, and 4-H1-anti-HSA-4-H1) were immobilized via amine coupling. Briefly, CM5 sensor chips were activated with a 1:1 mixture of 0.4 M 1-ethyl-3-(3-dimethylaminopropyl)carbodiimide (EDC) and 0.1 M N-hydroxysuccinimide (NHS), followed by injection of ligands in 10 mM sodium acetate (pH optimized individually) for 420 s. Remaining active groups were blocked with 1 M ethanolamine-HCl for 420 s (pH 8.0). Analytes (wild-type and mutated DS-Cav1, HSA, MSA) were prepared in running buffer (PBS containing 0.005% Tween-20) and injected over the sensor surface. The association phase was monitored for 100 or 400 s, followed by a 200 or 600 s dissociation phase in running buffer. Sensor surfaces were regenerated with 10 mM glycine-HCl (pH 2.0). Data were processed, and kinetic parameters were calculated using Biacore T200 evaluation software. All supplies, instrumentation, and analytical software were sourced from Cytiva unless otherwise specified.

### Plaque reduction neutralization test (PRNT)

Antibody neutralizing activity was assessed by PRNT using HEp-2 cells. Cells were detached by trypsinization and seeded in 96-well plates at 1.5 × 10⁴ cells/well 24 h prior to infection. Serially diluted antibodies in serum-free DMEM were mixed 1:1 (vol/vol) with RSV inoculum (also prepared in serum-free DMEM) and incubated at 37°C for 1 h. After removing the culture medium, 50 µL of the antibody-virus mixture was added to the cell monolayers and incubated at 37°C for 1 h. Unbound material was aspirated, and the monolayers were overlaid with semi-solid MEM. Following a 72 h incubation, the cells were fixed with paraformaldehyde and stained with the RB1 mAb specific for RSV F. After washing, HRP-conjugated goat anti-human IgG was applied. Plaques were visualized using TrueBlue Peroxidase Substrate and counted using ImmunoSpot Analyzer. The half-maximal inhibitory concentration (IC₅₀) was calculated by four-parameter logistic regression in GraphPad Prism 9.5.0, defined as the antibody concentration reducing plaque counts by 50% relative to virus-only controls.

### Epitope binning by bio-layer interferometry (BLI)

Epitope binning analyses were performed using a ForteBio Octet instrument equipped with Ni-NTA biosensors (ForteBio). DS-Cav1 antigen was diluted to 10 µg/mL in 0.02% PBST buffer (PBS containing 0.02% Tween-20). Fc-conjugated nanobodies and mAbs targeting distinct epitopes were prepared at final concentrations of 500 nM or 1 µM. Prior to the assay, biosensors were pre-wetted in PBST for 10 min. An initial baseline was taken in PBST (60 s), followed by antigen loading with 10 µg/mL DS-Cav1 (60 s). A post-loading baseline was recorded in PBST (60 s). Association kinetics were measured by exposing antigen-loaded biosensors to nanobody-Fc solution for 150–250 s. For competition assessment, the sensors bearing antigen-nanobody complexes were immersed in solutions containing mAbs targeting distinct known epitopes for 100–200 s. The reverse binding order was also tested, in which sensors were first exposed to competitor mAbs and then to nanobody-Fc. Biosensors were regenerated by three cycles, each consisting of a 5 s immersion in 500 mM imidazole and a 5 s immersion in PBST. Percentage inhibition was calculated as inhibition (%) =100 × [1 − (response with competitor mAb/response with isotype control)], where response denotes the binding signal (nm). Data analysis was performed using GraphPad Prism software.

### Computer-guided epitope mapping of RSV F protein

The interaction mechanism between 4-H1 and DS-Cav1 was investigated through integrated computational approaches. Homology modeling of 4-H1 was performed using Insight II 2000 (MSI Co.) based on its amino acid sequence, with explicit definition of framework regions (FRs) and CDRs. The model was optimized by energy minimization under a consistent valence force field (CVFF) via sequential steepest descent and conjugate gradient algorithms, followed by geometric validation via Ramachandran plot analysis. The DS-Cav1 structure was predicted with AlphaFold 3 and subjected to limited energy minimization under CVFF to relieve steric clashes. Molecular docking simulations employed the crystal structure of the DS-Cav1-D25 complex (PDB: 4JHA) as a topological template to predict binding modes for the 4-H1–DS-Cav1 complex. Using the refined complex structure, 50-ns molecular dynamics simulations were conducted with the Discovery Studio 3.5 module under CVFF to sample conformational space. All calculations were performed on IBM workstations using Insight II 2000 and Discovery Studio suites.

### Pharmacokinetic evaluation of 4-H1-anti-HSA-4-H1 in mice

To extend the half-life of 4-H1, we engineered a trivalent construct, 4-H1-anti-HSA-4-H1, by fusing two 4-H1 copies to an anti-HSA nanobody using (GS)₆ linkers. As a control, a 4-H1 trimer consisting of three 4-H1 copies linked in the same manner was prepared in parallel. The anti-HSA nanobody was developed through alpaca immunization and phage display screening against HSA. All constructs contained an N-terminal 6 × His tag for purification. After cloning into pcDNA3.1(+) vectors, constructs were expressed in 293 F cells and purified by Ni-NTA affinity chromatography. For *in vivo* evaluation, 6- to 8-week-old female BALB/c mice (Guangdong Yaokang Biotechnology Co., Ltd.) were randomly assigned to two groups (*n* = 3) and administered intranasally at a dose of 2 mg/kg of either 4-H1-anti-HSA-4-H1 or 4-H1-trimer. Blood samples were collected via retro-orbital puncture at 4, 24, 48, 96, 168, and 240 h post-administration. Serum was isolated by allowing blood to clot at RT for 4 h, followed by centrifugation at 2,000 × *g* for 15 min at 4°C. Serum nanobody concentrations were quantified by sandwich ELISA. Diluted serum samples were added to DS-Cav1-coated plates, and nanobody concentrations were quantified against standard curves generated with purified nanobodies. Serum concentration-time data were analyzed by non-linear regression fitting using GraphPad Prism version 9.5.0. The data were fitted to a constrained one-phase decay model defined by the equation: Y = (Y0 - Plateau)exp(-KX) + Plateau, where Y0 is the initial concentration, K is the rate constant, and the Plateau was constrained to zero. The elimination half-life (T_1/2_) was calculated as ln (2)/K. The area under the curve (AUC) was calculated using the trapezoidal rule, and the maximum serum concentration (Cmax) was determined directly from the observed concentration-time data.

### *In vivo* efficacy of 4-H1-anti-HSA-4-H1 against RSV in mice

The *in vivo* prophylactic efficacy of the bispecific 4-H1-anti-HSA-4-H1 against RSV was evaluated in 6-week-old female BALB/c mice (*n* = 6). Mice were randomly divided into two groups and administered 2 mg/kg 4-H1-anti-HSA-4-H1 or PBS vehicle control intranasally in a 50 µL volume 24 h prior to viral challenge. Following compound delivery, mice were anesthetized with isoflurane and inoculated intranasally with 4 × 10⁶ PFU of RSV A2 in 50 µL PBS. Lungs were harvested at 4 days post-infection for viral load quantification and histopathological analysis.

### H&E staining of RSV-infected cells in tissues

Resected left lung tissue specimens were fixed in 4% paraformaldehyde at RT for 24 h to preserve morphological integrity. Following fixation, tissues were dehydrated through a graded ethanol series, cleared in xylene, and then embedded in paraffin blocks. Serial sections of 5 µm thickness were cut using a precision microtome (Leica). For histological evaluation, sections were stained with H&E (Servicebio, G1076) according to standardized protocols. Histopathological scoring was evaluated as described in the study (39, 40).

### Viral load measurement by quantitative reverse transcription PCR (RT-qPCR)

RNA extraction from right lung tissues was performed using the EZBio Viral RNA Kit (EZBioscience, EZB-VRN1). Prior to extraction, tissues were mechanically homogenized in a Freezer Mill (Guangzhou Luka Sequencing Instrument Co., Ltd., Models LUKYM-I/II/III) under the following conditions: −35°C (the actual temperature reached −65°C during operation), 70 Hz, for 150 s, repeated twice. First-strand cDNA was then synthesized from the extracted RNA using HiScript III Reverse Transcriptase (Vazyme, R312). RT-qPCR reactions (20 µL final volume) contained 2 µL of cDNA template, 10 µL of ChamQ SYBR qPCR Master Mix (Vazyme, Q311), and 0.4 µM primers against the RSV nucleoprotein (NP) gene (GenBank accession no. NC_ 038235.1) (Forward: 5′-AGATCAACTTCTGTCATCCAGCAA-3′, Reverse: 5′-AACATGCCACATAACTTATTGAT-3′). Amplification was conducted on an ABI 7900HT system (Applied Biosystems) under standardized cycling conditions. Viral NP RNA levels were normalized to endogenous mRPL13A mRNA expression using the 2⁻ΔΔCt method.

### Statistical analyses

The graphical and statistical evaluations were conducted using Prism software (GraphPad Prism 9.5.0). The data are presented as the mean ± standard error of the mean. Comparisons between experimental and control groups were made using unpaired two-tailed *t*-tests. Statistical significance was defined as *P* values < 0.05.

## RESULTS

### DS-Cav1 immunization elicits serum with potent neutralizing activity against RSV

**Fig 1:**
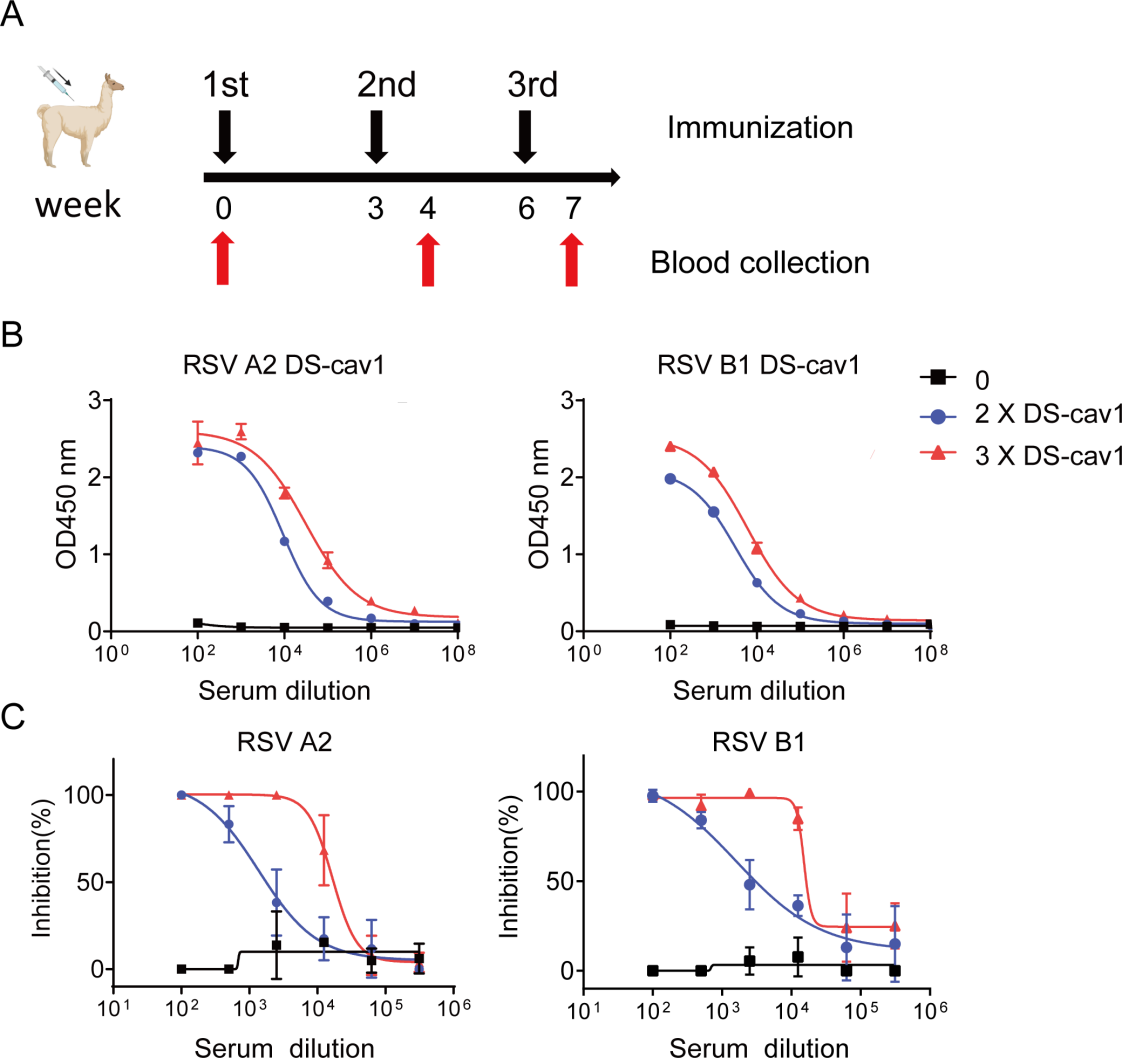
Characterization of serum from DS-cav1-immunized alpaca. (**A**) Experimental immunization schedule. Serum samples, collected from an alpaca immunized with RSV A2 DS-cav1 protein at three time points (pre-immunization, and after the second and third immunizations), were analyzed for binding (**B**) and neutralizing activity (**C**). In (**B**), binding to RSV A2 (left panel) and RSV B1 (right panel), DS-cav1 was assessed by ELISA. The y-axis represents absorbance at 450 nm, whereas the x-axis indicates serum dilution folds. In (**C**), neutralizing activity against RSV A2 (left panel) and RSV B1 (right panel) was measured by PRNT. The y-axis shows percent neutralization, and the x-axis indicates serum dilution folds. For both (**B**) and (**C**), data points represent the mean of two technical replicates, and error bars indicate the standard deviation (SD). The blue curve (“2 × DS-cav1”) and red curve (“3 × DS-cav1”) correspond to serum collected one week after the second and third immunizations, respectively. The black curve (“0”) represents pre-immunization serum.

Based on the enhanced neutralizing potency of antibodies targeting pre-F-specific epitopes, we generated the pre-F-stabilized RSV F variant DS-Cav1, incorporating the stabilizing mutations and a C-terminal “foldon” trimerization domain (38). An alpaca was immunized with eukaryotic-expressed DS-Cav1 at weeks 0, 3, and 6 (Fig. 1A). Serum collected after both second and third immunizations exhibited high nanobody titers against RSV A2 and B1 DS-Cav1 antigens, confirming robust humoral immunity (Fig. 1B). Compared with post-second immunization samples, serum from the third immunization demonstrated significantly enhanced neutralizing activity against RSV A2 and B1, achieving ∼10-fold higher 50% inhibitory dilution (ID₅₀) titers for both strains (Fig. 1C). These results establish that DS-Cav1 immunization elicits potent neutralizing antibody responses against both RSV subtypes, providing a solid foundation for isolating anti-RSV neutralizing nanobodies that neutralize both RSV subgroups.

### Isolation and characterization of anti-RSV F nanobodies

**Fig 2:**
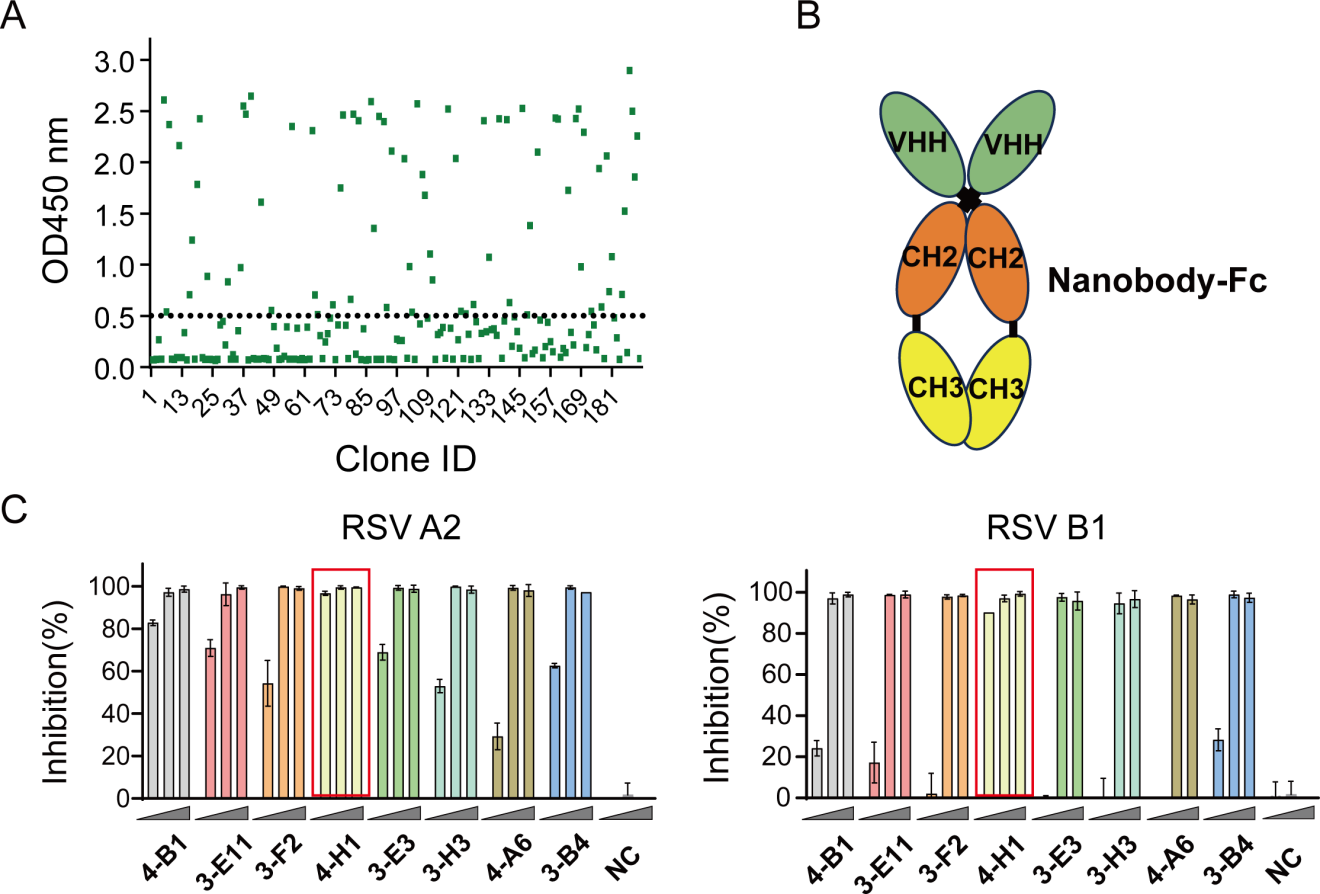
Isolation and characterization of anti-RSV F nanobodies. (**A**) The binding capacity of phage supernatants (96 clones per round from rounds 3 and 4) to RSV A2 DS-cav1 was determined by phage ELISA. Each dot corresponds to a single clone tested. (**B**) Schematic representation of the nanobody-Fc construct. Nanobody genetically fused to the human IgG1 Fc fragment (CH2–CH3). (**C**) Neutralizing activity of nine purified Fc-fused nanobodies against RSV A2 (left panel) and RSV B1 (right panel) measured by PRNT. The y-axis represents percent neutralization, and arrows indicate tested concentrations of nine purified Fc-fused nanobodies (0.02, 0.2, 2 µg/mL), NC refers to the negative control nanobody.

PBMCs were isolated for the construction of a phage display library to screen for nanobodies. Biotinylated DS-Cav1 was immobilized on streptavidin-coated magnetic beads via biotin-streptavidin conjugation to preserve conformational integrity. In rounds 1 and 2, phage pools were directly panned against immobilized DS-Cav1 to enrich binders. Beginning in round 3, phage libraries were pre-incubated with a cocktail of site I-IV antibodies before antigen exposure. This competitive blocking step depleted binders to low-neutralization epitopes, resulting in preferential enrichment of clones recognizing high-neutralization-potency epitopes. Following four rounds of biopanning, single phage clones from the enriched library were screened by phage ELISA. The results showed that 41 out of 96 clones from the third round and 32 out of 96 clones from the fourth round were positive binders (Fig. 2A). Based on ELISA signals and sequence analysis, eight nanobodies were selected for further characterization. These nanobodies were expressed as nanobody-human IgG1 Fc fusion proteins in 293 F cells (Fig. 2B). All eight nanobodies exhibited neutralizing activity against RSV infection, with 4-H1-Fc showing the highest potency against both subtypes (Fig. 2C). Subsequent bio-layer interferometry (BLI)-based mAb competition assays revealed that all nanobodies competed with D25 (the precursor to nirsevimab targeting site Ø) for antigen binding (41). Binding curves demonstrated that pre-incubation of DS-Cav1 with any selected nanobodies inhibited D25 binding. In contrast, pre-saturation of DS-Cav1 with the non-neutralizing antibody, referred to as NC, did not impair D25 binding (Fig. S1). Overall, these findings demonstrate that our biopanning strategy successfully isolated potent neutralizing nanobodies targeting site Ø.

### Characterization of 4-H1

**Fig 3:**
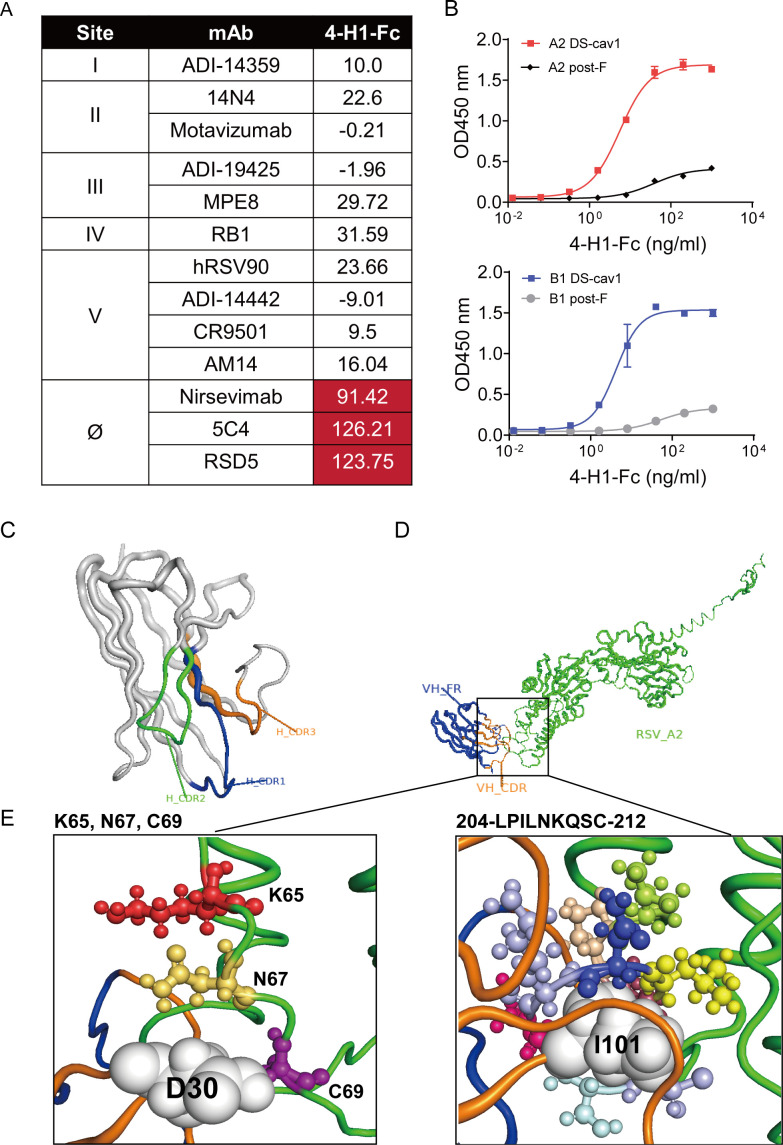
Binding activity of 4-H1 to RSV F and its epitopes. (**A**) Epitope mapping via BLI-based cross-competition assays. RSV A2 DS-cav1 protein was immobilized on Ni-NTA biosensors. Biosensors were exposed to competitor antibodies followed by 4-H1-Fc. Percent inhibition was calculated by comparing the binding response maxima of 4-H1-Fc in the absence and presence of each competitor. (**B**) The binding capacity of 4-H1-Fc to F protein was determined by ELISA. (**C**) The 3D ribbon structures of the 4-H1 fragment. The deep blue ribbon denotes CDR1, the green denotes CDR2, and the orange denotes CDR3. (**D**) Modeled complex of 4-H1 and RSV A2 DS-cav1. The green ribbon denotes the orientation of the DS-cav1 fragment, the orange denotes 4-H1 CDRs, and the deep blue denotes 4-H1 FRs. (**E**) Key interacting amino acid residues of DS-cav1 identified by theoretical analysis. The white sphere models represent amino acids of 4-H1 (D30 AND I101), the ball-and-stick models show amino acids of DS-cav1 (K65, N67, C69, and the sequence 204-LPILNKQSC-212).

Based on superior neutralizing potency against RSV A2 and B1, 4-H1 was selected for further characterization (Fig. 2C). Epitope mapping via BLI-based cross-competition assays using site-specific mAbs revealed that pre-saturation of DS-Cav1 with site Ø mAbs (nirsevimab, 5C4, RSD5 [12, 42, 43]) abolished subsequent 4-H1-Fc binding. In contrast, mAbs targeting sites I (ADI-14359 [44]), II (motavizumab, 14N4 [45, 46]), III (ADI-19425, MPE8 [44, 47]), IV (RB1 [48]), and V (CR9501, ADI-14442, hRSV90, AM14 [49–52]) showed minimal competition, confirming exclusive specificity of 4-H1 for site Ø (Fig. 3A). Given the site Ø specificity observed by BLI, we assessed the conformational dependence of 4-H1 by ELISA. 4-H1-Fc demonstrated potent binding to pre-F (DS-Cav1, EC₅₀ =5.575 ng/mL for RSV A2 and 4.188 ng/mL for RSV B1) but minimal reactivity toward post-F, confirming preferential recognition of the pre-F conformation (Fig. 3B). For structural modeling, energy minimization was performed using the consistent valence force field (CVFF). The 4-H1 framework underwent 30,000 iterations with steepest descent followed by conjugate gradient algorithms, converging at an energy gradient threshold of 0.02 kcal/mol (Fig. 3C). Ramachandran analysis confirmed >99% of residues in allowed regions. DS-Cav1 was refined identically. Using the experimental DS-Cav1-D25 complex as a topological scaffold, the theoretical 3D complex structure of 4-H1 and DS-Cav1 was generated by constrained docking methods (Fig. 3D). Analysis of interactions within a 0.6 nm radius of 4-H1 CDRs, including van der Waals forces, hydrogen bonds, polar contacts, and electrostatic potentials, revealed two high-affinity binding clusters on DS-Cav1, that is, residues 65/67/69 and 204-212, respectively (Fig. 3E). The spatial adjacency of these clusters to critical binding residues of established site Ø mAbs (nirsevimab, RSD5, 5C4) suggests potential overlap in their binding interfaces (12, 42, 43).

### Epitope mapping of RSV F bound to 4-H1

**Fig 4:**
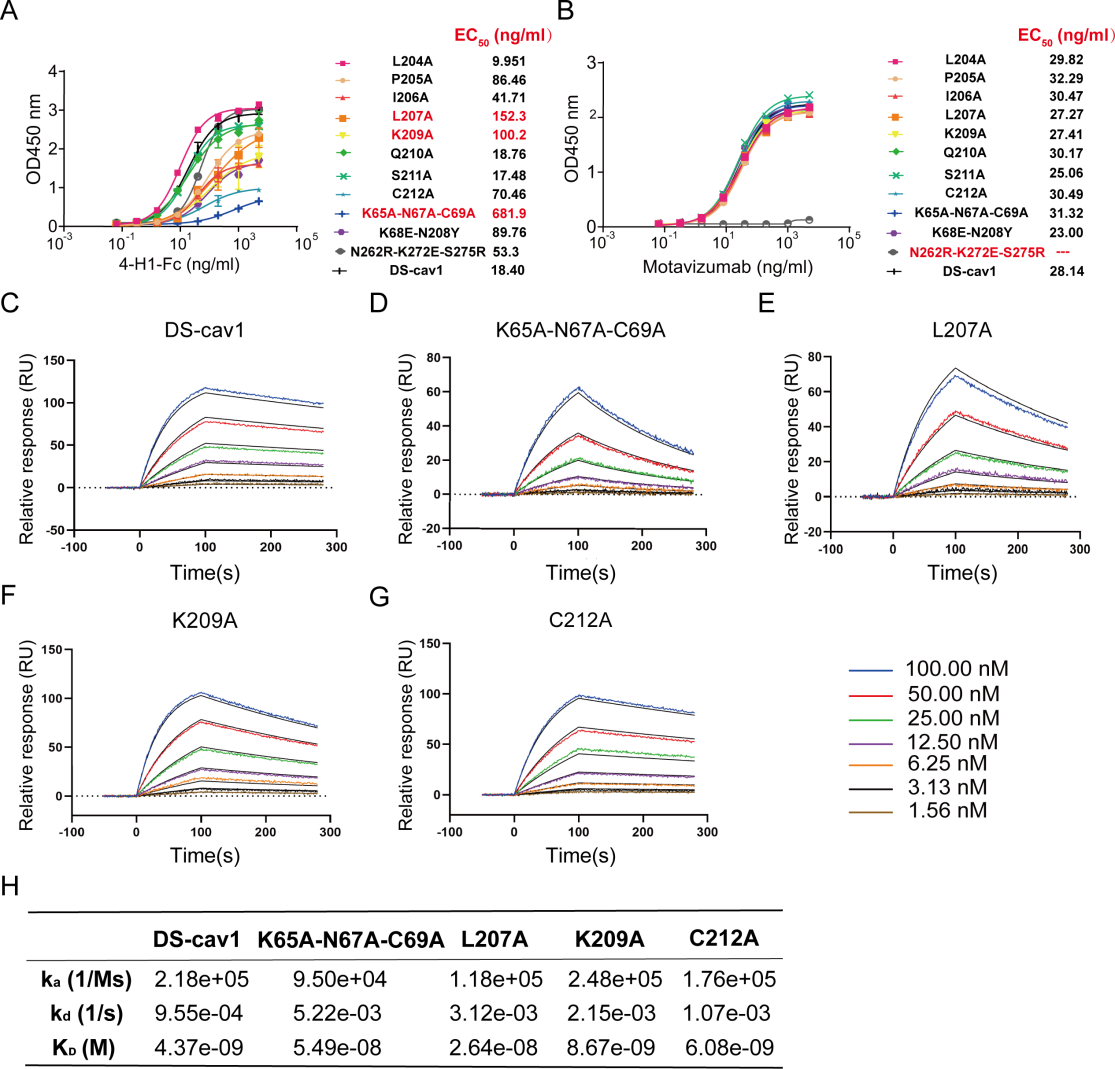
4-H1 epitope identification. Binding profiles of 4-H1-Fc (**A**) and motavizumab (**B**) against RSV A2 wild-type and mutated DS-cav1. The EC_50_ values for the binding of each mutated DS-cav1 are listed on the right side of the panel. Residues causing a greater than 5-fold increase in EC₅₀, along with their corresponding values, were highlighted in red. “---” indicates no binding. SPR sensorgrams showing binding of wild-type (**C**), K65A-N67A-C69A (**D**), L207A (**E**), K209A (**F**), and C212 (**G**) mutated DS-cav1 to immobilized 4-H1. Analytes were injected in 2-fold serial dilutions ranging from 100 nM to 1.56 nM. (**H**) Summary of binding kinetics (association rate [ka], dissociation rate [kd], and equilibrium dissociation constant [K_D_]) determined by SPR.

To map functional residues, we performed alanine scanning mutagenesis on DS-Cav1 at positions L204, P205, I206, L207, K209, Q210, S211, and C212, based on the volume and character of amino acid residues. We also generated the K65A-N67A-C69A triple-mutated protein. Control antigens included DS-Cav1 variants encoding the F protein sequences from a nirsevimab-escape mutant (harboring K68E-N208Y substitutions) and a palivizumab binding-attenuated mutant (carrying N262R-K272E-S275R mutations) (39, 45). ELISA screening at 2 µg/mL coating concentration revealed that only K65A-N67A-C69A exhibited marginally reduced binding to 4-H1-Fc. By contrast, this mutation severely impaired 5C4 and RSD5 binding, whereas nirsevimab binding remained unaffected. Importantly, both 4-H1-Fc and RSD5 retained binding to the K68E-N208Y, suggesting their epitope divergence with nirsevimab and 5C4 (Fig. S2A). When antigen coating was reduced to 0.5 µg/mL to enhance sensitivity, the L207A, K209A, and K65A-N67C-C69A mutations disrupted 4-H1-Fc binding, resulting in EC₅₀ values > 100 ng/mL. In contrast, motavizumab binding remained unaffected under these conditions (Fig. 4A and B).

To precisely define the binding kinetics underlying these disruptions, we performed SPR analysis. Using His-tagged 4-H1, we observed that compared with wild-type DS-Cav1, the L207 and K65A-N67A-C69A mutated DS-Cav1 showed reduced association rates (k_a_, 1.18 × 10⁵/Ms and 9.50 × 10^4^ /Ms) and increased dissociation rates (k_d_, 3.12 × 10⁻^3^/s and 5.22 × 10⁻^3^/s) to 4-H1, resulting in K_D_ values of 26.4 nM and 54.9 nM, respectively (Fig. 4D and E). This represents a ∼6-fold and 13-fold affinity decrease relative to wild-type (K_D_ = 4.37 nM, Fig. 4C). The K209A mutated protein displayed mild affinity loss (K_D_ = 8.67 nM) solely through increased k_d_ (2.15 × 10⁻^3^/s) (Fig. 4F). These SPR results correlated with ELISA EC₅₀ data. Additionally, the C212 structural disulfide bond stabilizes F protein folding, but we found it did not contribute to functional epitope-paratope interactions, as evidenced by minimal changes in the binding kinetics of C212A despite reduced ELISA signals (Fig. 4G). All SPR binding kinetics parameters are listed in Fig. 4H. Structural modeling quantified atomic distances between paratope residues (D30, I101) and antigenic site Ø residues (K65, N67, C69, L207, and K209). Specific atoms within the side chains of C69, L207, and K209 were positioned within 3 Å of D30 or I101, suggesting potential hydrogen bonding or hydrophobic interactions. In contrast, K65 and N67 exhibited longer interaction distances, indicating weaker energetic contributions to complex stabilization (Fig. S2B). Collectively, these data define a shared dependency on the K65-N67-C69 cluster among 5C4, RSD5, and 4-H1, indicating that this cluster constitutes a critical component of their overlapping functional epitopes. In contrast, nirsevimab binding is independent of this cluster. Although 4-H1 and nirsevimab share no critical binding residues, their epitopes spatially neighbor each other (12), suggesting that steric hindrance, not epitope identity, may mediate the observed complete competition.

### 4-H1-anti-HSA-4-H1, heterotrimeric and bispecific for F and HSA, exhibits robust neutralization against RSV

**Fig 5:**
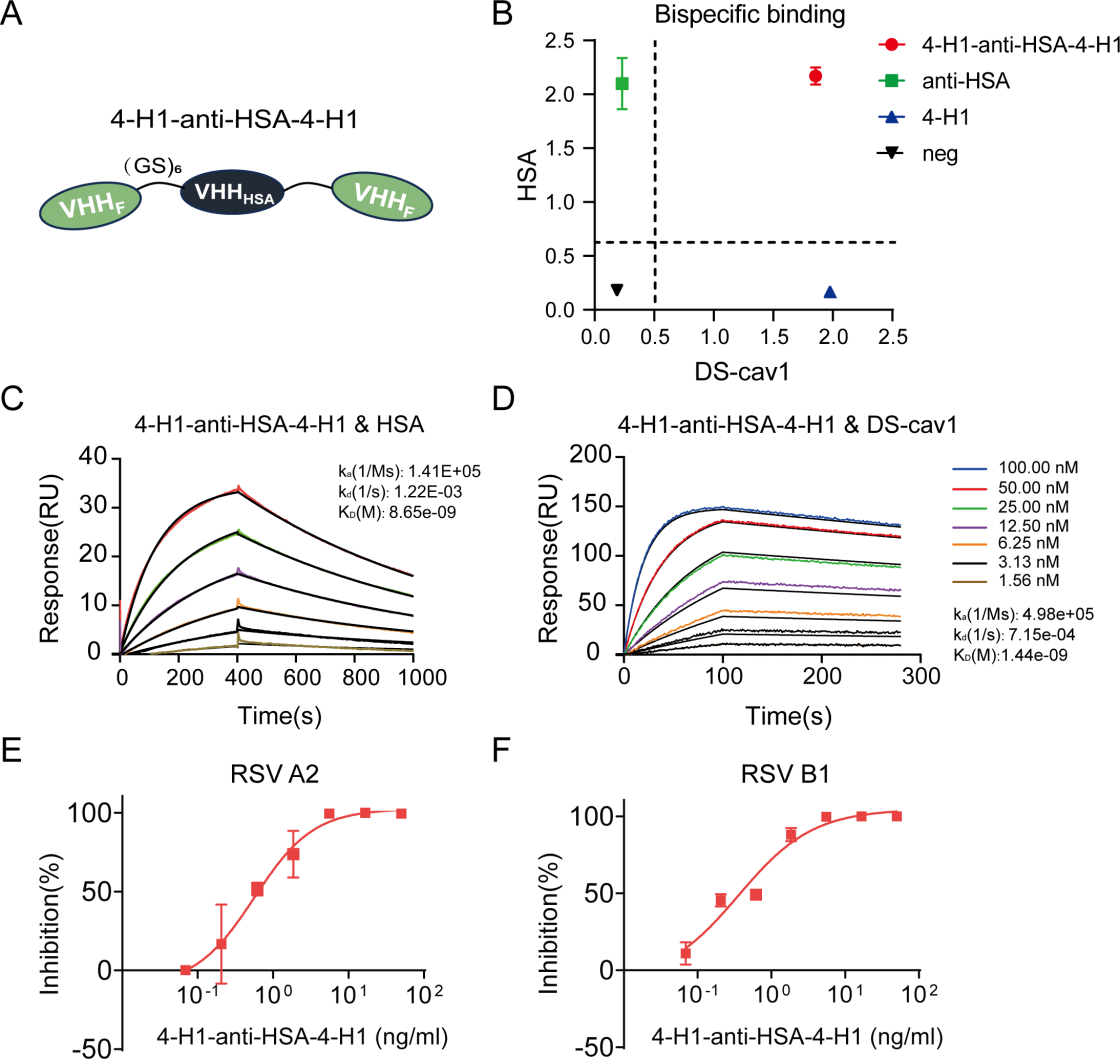
Design and characterization of bispecific nanobody. (**A**) Schematic of engineered nanobody formats. (**B**) Binding capacity of nanobodies to RSV A2 DS-cav1 and HSA determined by ELISA. The x-axis shows absorbance at 450 nm for DS-cav1 binding, and the y-axis shows absorbance at 450 nm for HSA binding. SPR sensorgrams showing binding of HSA (**C**) and RSV A2 DS-cav1 (**D**) to immobilized 4-H1-anti-HSA-4-H1. Analytes were injected in 2-fold serial dilutions ranging from 100 nM (DS-cav1) or 50 nM (HSA) to 1.56 nM. Neutralizing activity of 4-H1-anti-HSA-4-H1 against RSV A2 (**E**) and RSV B1 (**F**) determined by PRNT. The y-axis shows percent neutralization, and the x-axis shows 4-H1-anti-HSA-4-H1 concentration.

To enhance the *in vivo* efficacy and stability of 4-H1, we engineered a heterotrimeric bispecific construct, 4-H1-anti-HSA-4-H1. This molecule fuses two 4-H1 copies to a novel anti-HSA nanobody via (GS)₆ linkers (Fig. 5A).

The underlying design principle is supported by a prior study in which an HSA-fused construct demonstrated longer serum half-life and higher peak serum concentration (Cmax) than the 3 × Nb15 control. Notably, it achieved effective lung delivery with sustained localization after intranasal administration, providing significant protection against SARS-CoV-2 when administered 24 h pre-exposure (53).

The novel anti-HSA nanobody, developed in our laboratory, exhibits a binding affinity for HSA of 5.47 nM (Fig. S3A) and cross-reacts with mouse serum albumin (MSA) (Fig. S3B). ELISA confirmed that this construct binds simultaneously to both HSA and DS-Cav1 (Fig. 5B). Building on these results, SPR assays were subsequently performed to characterize binding alterations resulting from dual 4-H1 integration.

Assessment of 4-H1-anti-HSA-4-H1 binding showed a modest decrease in HSA affinity (K_D_ = 8.65 nM, Fig. 5C) compared with the monomeric anti-HSA control (5.47 nM, Fig. S3A). Conversely, DS-Cav1 binding was enhanced (K_D_ = 1.44 nM versus 4.37 nM for monomeric 4-H1, Fig. 5D and 4C). This enhancement in DS-Cav1 affinity may stem from the bivalently bound avidity facilitated by the dual 4-H1 architecture, potentially stabilizing the antigen-antibody complex and increasing functional valency. Neutralization capacity assessed via PRNT demonstrated exceptional potency against both RSV subtypes, with IC_50_ values of 0.5641 ng/mL for A2 and 0.3571 ng/mL for B1, as shown in Fig. 5E and F.

**Fig 6:**
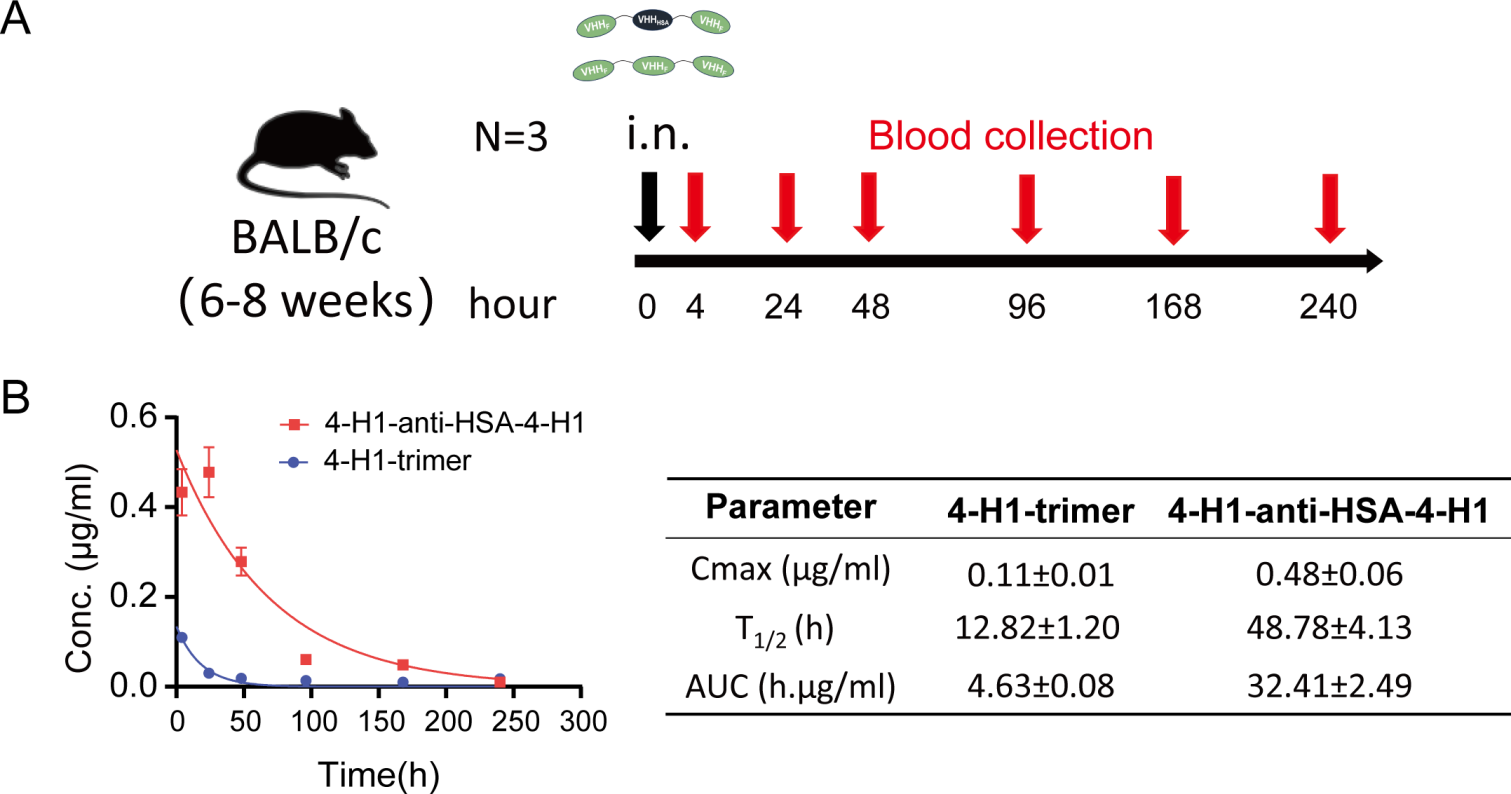
Pharmacokinetics of 4-H1-anti-HSA-4-H1 and 4-H1 trimer *in vivo*. (**A**) Experimental timeline. BALB/c mice were administered 4-H1-anti-HSA-4-H1 or 4-H1-trimer intranasally (i.n.) at 0 h. Blood samples were collected at the indicated time points for pharmacokinetic analysis. (**B**) Serum concentration–time profiles following intranasal administration. Serum concentrations (y-axis) are plotted against time (x-axis) for 4-H1-anti-HSA-4-H1 and 4-H1-trimer. The solid lines represent the pharmacokinetic curves generated by fitting the data to a constrained one-phase decay model, which resulted in R² values of 0.8838 for 4-H1-anti-HSA-4-H1 and 0.8783 for 4-H1-trimer. The accompanying table summarizes the mean ± SD values of Cmax, T₁/₂, and AUC for each construct (*n* = 3 mice).

Building on the potent *in vitro* neutralization activity of 4-H1-anti-HSA-4-H1, we evaluated its pharmacokinetics following intranasal administration in mice, using a monospecific 4-H1 trimer (lacking anti-HSA) as a control (Fig. 6A). Blood samples were obtained at six post-administration time points (4, 24, 48, 96, 168, and 240 h) for serum isolation, and the nanobody concentrations were subsequently quantified via the developed sandwich ELISA. Our analysis revealed that 4-H1-anti-HSA-4-H1 exhibits significantly extended serum persistence, demonstrating a half-life (T_1/2_) of 48.78 h versus 12.82 h for the control trimer, a ∼4-fold higher Cmax, and a ∼7-fold greater serum area under the curve (AUC, Fig. 6B). These enhanced pharmacokinetic properties suggest enhanced retention in respiratory mucosa, supporting its progression to challenge models based on robust neutralization capacity and favorable pharmacokinetics.

### *In vivo* anti-RSV activity of 4-H1-anti-HSA-4-H1

**Fig 7:**
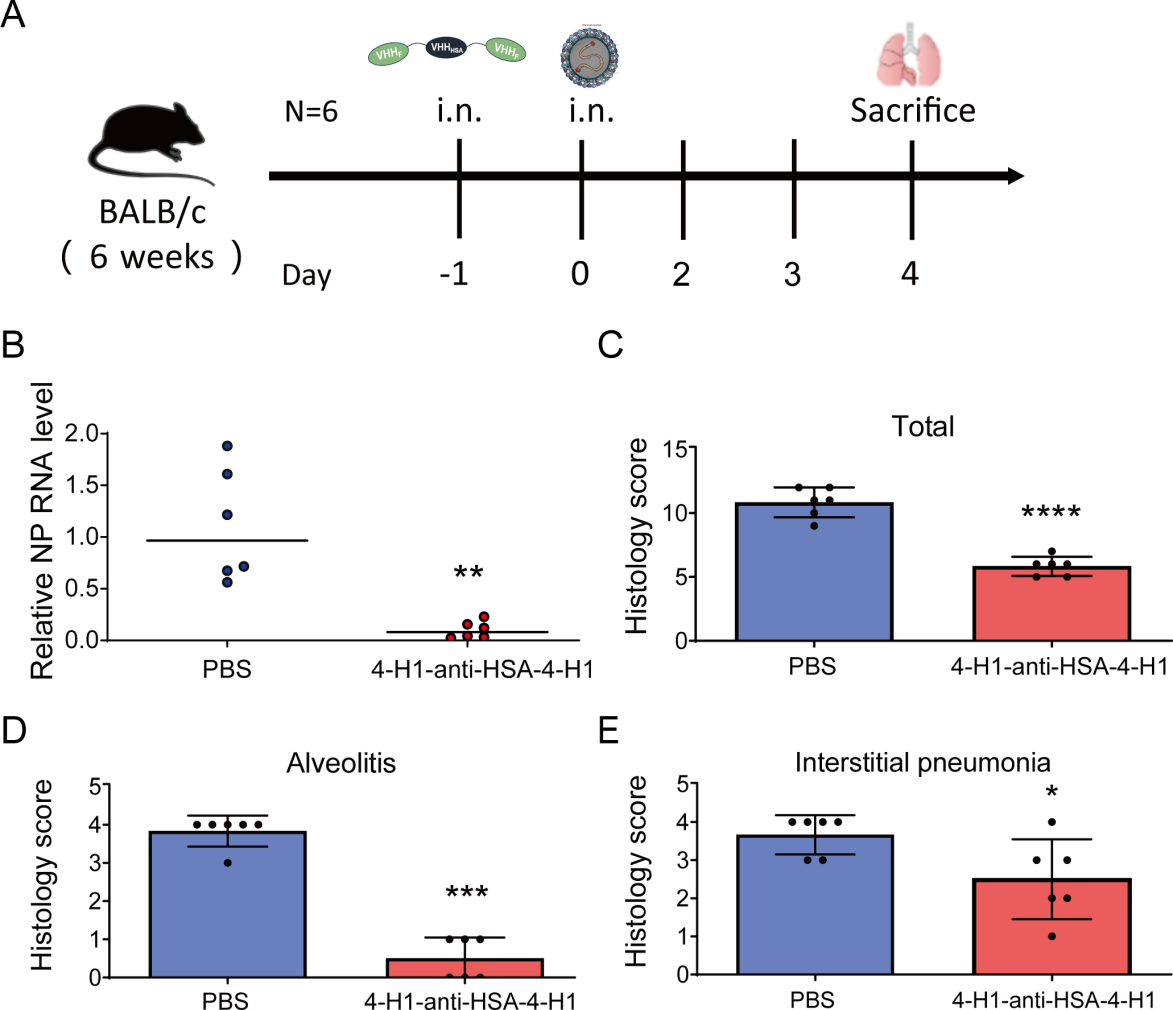
The efficacy of 4-H1-anti-HSA-4-H1 evaluated in mice challenged by RSV A2. (**A**) Experimental design for *in vivo* efficacy. (**B**) Four days after infection, pulmonary RSV load was determined by RT-qPCR quantification of relative NP RNA levels, normalized to endogenous mRPL13A. Quantitative histopathology scoring of murine lungs assessed in a blinded manner for combined pathology (**C**), alveolitis (**D**), and interstitial pneumonia (**E**). The pathology scoring system assigned 0-4 points to each lung section based on inflammatory cell infiltration levels in various lung tissue compartments (*P < 0.05, **P < 0.01, ***P < 0.001, ****P < 0.0001).

Finally, we evaluated the *in vivo* antiviral efficacy of 4-H1-anti-HSA-4-H1 against RSV in mice. Animals received intranasal administration of 2 mg/kg 4-H1-anti-HSA-4-H1 or an equal volume of PBS, followed by intranasal challenge with 4 × 10⁶ PFU RSV A2 24 h later (Fig. 7A). Four days after the challenge, lungs were harvested for viral titer determination and histopathological examination. Viral RNA was quantified by an RSV NP-specific RT-qPCR, and the lungs of mice treated with 4-H1-anti-HSA-4-H1 were borderline positive for RSV RNA, whereas the relative amount of RNA indicated much higher viral load in samples from PBS-treated mice (Fig. 7B). Next, we performed H&E staining to assess viral lung pathology. The PBS-treated mice exhibited severe pulmonary inflammation, characterized by inflammatory infiltration across multiple tissue compartments. In contrast, a mild lung infiltration was observed in the prophylactic 4-H1-anti-HSA-4-H1 treated group (Fig. S4). Quantitative pathology scoring confirmed markedly lower injury in the 4-H1-anti-HSA-4-H1 treated group (Fig. 7C), with significantly reduced alveolitis and interstitial pneumonia (Fig. 7D and E). Collectively, these findings demonstrate that 4-H1-anti-HSA-4-H1 significantly mitigated RSV-induced pulmonary damage, indicating its value as a prophylactic candidate against RSV infection.

## DISCUSSION

RSV imposes a substantial global burden of severe respiratory disease (54–56). To develop high-potency therapeutics, we pursue molecules with exceptional neutralizing activity against RSV. Given the documented superiority of pre-F-specific antibodies over those targeting epitopes present in both pre-F and post-F conformations (57), an alpaca was immunized with the engineered pre-F-stabilized F trimer DS-Cav1. Through an epitope-blocking depletion strategy, we isolated a panel of site Ø-directed nanobodies, with 4-H1 exhibiting the most potent inhibitory activity. Critically, epitope characterization established that 4-H1 engages site Ø residues K209, C212, and the K65-N67-C69 cluster, as demonstrated by impaired binding upon alanine substitution. Notably, in ELISA binding assays at 2 µg/mL antigen coating, none of the mutated proteins markedly impaired 4-H1-Fc binding. By contrast, other site Ø-directed mAbs showed substantial binding reduction for specific mutations. Smaller binding perturbation by point mutations suggests distributed energy contributions across the epitope. We propose that 4-H1 recognizes an epitope with remarkable mutational resilience, requiring multiple substitutions for escape. Notably, no site V-targeting antibodies were isolated during initial screening. We attribute this to steric occlusion by prebound site I-IV antibodies, which may have physically masked site V through spatial overlap or induced conformational masking via allosteric effects. Additional unidentified factors could also contribute to the absence of site V binders. In subsequent efforts, we will employ post-F negative selection to replace the epitope-blocking strategy, thereby potentially enabling isolation of site V-specific binders. Following successful isolation, biparatopic agents incorporating both site Ø- and V-targeting nanobodies will be developed. This design is expected to enhance neutralizing potency and limit viral escape.

Owing to their small size (~15 kDa), nanobodies undergo rapid renal clearance (58), necessitating half-life extension strategies. Albumin-binding approaches represent a clinically validated method, exemplified by the trivalent bispecific nanobody Ozoralizumab (59). This molecule incorporates two TNF-α-targeting domains and an HSA-binding module that extends half-life via neonatal Fc receptor (FcRn)-mediated recycling (59, 60). Despite multiple reported anti-RSV F nanobodies, none have achieved clinical approval (31, 33–37). Notably, ALX-0171—a trivalent anti-RSV F nanobody developed for therapeutic use—progressed to phase II trials but was terminated due to failure in reducing hospitalization duration(31). Other previously described nanobodies also exhibit certain limitations. For instance, pre-F-specific nanobodies such as m17 and m35 recognize a novel prefusion epitope, termed site VI, and effectively prevent conformational rearrangements during the fusion process. However, their neutralization potency against both RSV A and B subtypes is markedly less potent than that of other nanobodies (35). Another nanobody, F-VHHb, which targets regions IV–VI, has been shown to effectively inhibit viral entry and suppress replicative virus to undetectable levels in mice when administered prior to challenge. Nevertheless, its efficacy against RSV B was not evaluated, limiting the assessment of its broad-spectrum potential (34, 37). Similarly, F-VHH-Cl184, which primarily binds antigenic site I with additional interactions in sites III and IV, demonstrates markedly reduced neutralization against RSV B compared to RSV A (33). To our knowledge, this represents the first report of a pre-F-specific antibody predominantly targeting antigenic site I. In contrast, the pre-F-specific nanobody F-VHH-4, developed by the same team, exhibits superior neutralizing activity and is among the most potent nanobodies reported to date. It provides effective prophylaxis at doses as low as 0.5 mg/kg in mice. Structural studies reveal that F-VHH-4 targets a conserved cavity formed between two F protomers, engaging residues from antigenic sites II, III, IV, and V (36). However, its application is constrained by rapid clearance, necessitating administration shortly before viral exposure, as demonstrated in the referenced study, where it was administered 4 h prior to challenge (36). To address these limitations, we engineered heterotrimeric 4-H1-anti-HSA-4-H1, synergizing potent neutralization via site Ø with albumin-mediated half-life extension. This molecule potently neutralizes both RSV A2 and B1, exhibiting neutralizing activity that is comparable with, if not greater than, any nanobody reported to date. Leveraging albumin-mediated recycling, the construct achieved a 48.78 h serum half-life in mice, markedly surpassing that of the unconjugated control trimer (12.82 h). Consequently, a single intranasal administration (2 mg/kg) conferred robust protection, as evidenced by reduced lung viral load via RT-qPCR and attenuated lung pathology in H&E-stained sections.

Regrettably, no infectious viral titers were detected by plaque assay in any group. We reason that this is likely due to technical limitations in our sample processing protocol. The core issue appears to be that the entire workflow, in our hands, was suboptimal for preserving the infectivity of the labile RSV, with the intensive mechanical homogenization step being a potential critical point. Nevertheless, the liberated viral RNA remained quantifiable by RT-qPCR. Prior studies have validated RT-qPCR-based quantification of RSV in tissues (36, 61–65), with some relying on it without additional viral titrations of the lung (61, 63, 64), thus supporting its use as a reliable surrogate metric.

Critically, the < 45 kDa size enables efficient inhalation delivery, achieving high local drug concentrations at respiratory infection sites while minimizing systemic barriers. This confers potential for faster and more potent local antiviral effects compared with systemic delivery of the identical drug (66, 67). Such rapid efficacy is vital for RSV management, as most infected infants are hospitalized at or after peak viral replication and disease progression, missing the early intervention window. Hence, immediate respiratory mucosal drug delivery is essential to arrest pathogenesis. Accordingly, our future work will focus on formulating the 4-H1-anti-HSA-4-H1 molecule for nebulized delivery and rigorously evaluating its prophylactic and therapeutic potential in relevant respiratory infection models. Despite achieving an extension in serum half-life versus the control (48.78 h vs 12.82 h), this half-life remains insufficient for long-term prophylaxis. The reduced binding affinity of 4-H1-anti-HSA-4-H1 for MSA, as indicated by its ∼5-fold higher EC₅₀ against MSA than HSA (Fig. S3C), likely underlies this limitation. Beyond this, optimization of HSA binding affinity remains critical to enhance pharmacokinetic properties and enable robust clinical application.

In summary, respiratory-administered bispecific nanobodies represent a next-generation therapeutic platform. By integrating potent neutralization, extended durability, and targeted delivery, 4-H1-anti-HSA-4-H1 may provide a promising strategy for RSV prevention and treatment.

## Data Availability

The original contributions presented in the study are included in the article and supplemental material. Further inquiries can be directed to the corresponding authors.

## SUPPLEMENTAL MATERIAL

Figure S1 jvi.01285-25-s0001.tif Epitope mapping via BLI-based competition assays.

Figure S2 jvi.01285-25-s0002.tif 4-H1 epitope identification.

Figure S3 jvi.01285-25-s0003.tif Assessment of anti-HSA nanobody binding and cross-reactivity.

Figure S4 jvi.01285-25-s0004.tif H&E staining of lung tissue sections from mice treated with PBS or 4-H1-anti-HSA-4-H1.

Supplemental material jvi.01285-25-s0005.docx Table S1; legends for Fig. S1 to S4.

ASM does not own the copyrights to Supplemental Material that may be linked to, or accessed through, an article. The authors have granted ASM a non-exclusive, world-wide license to publish the Supplemental Material files. Please contact the corresponding author directly for reuse.
