# Supplementary figures and images for: A novel bispecific nanobody protects mice against RSV infection via intranasal administration
Source: J Virol. 2025 Nov 24;99(12):e01285-25. doi: 10.1128/jvi.01285-25 (PMC12724135; doi:10.1128/jvi.01285-25)

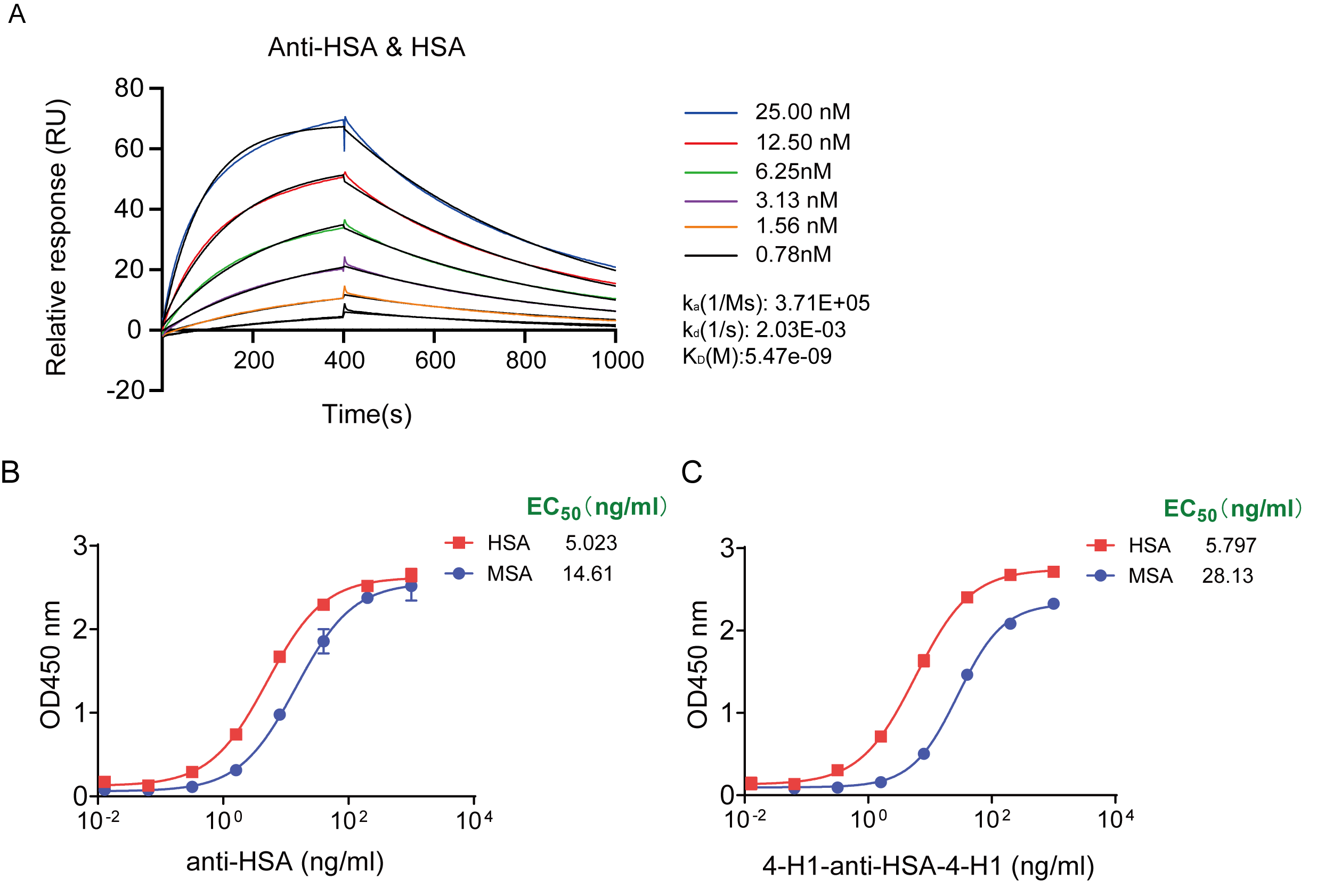

Supplement: Figure S1 — Epitope mapping via BLI-based competition assays. [file jvi.01285-25-s0001.tif]

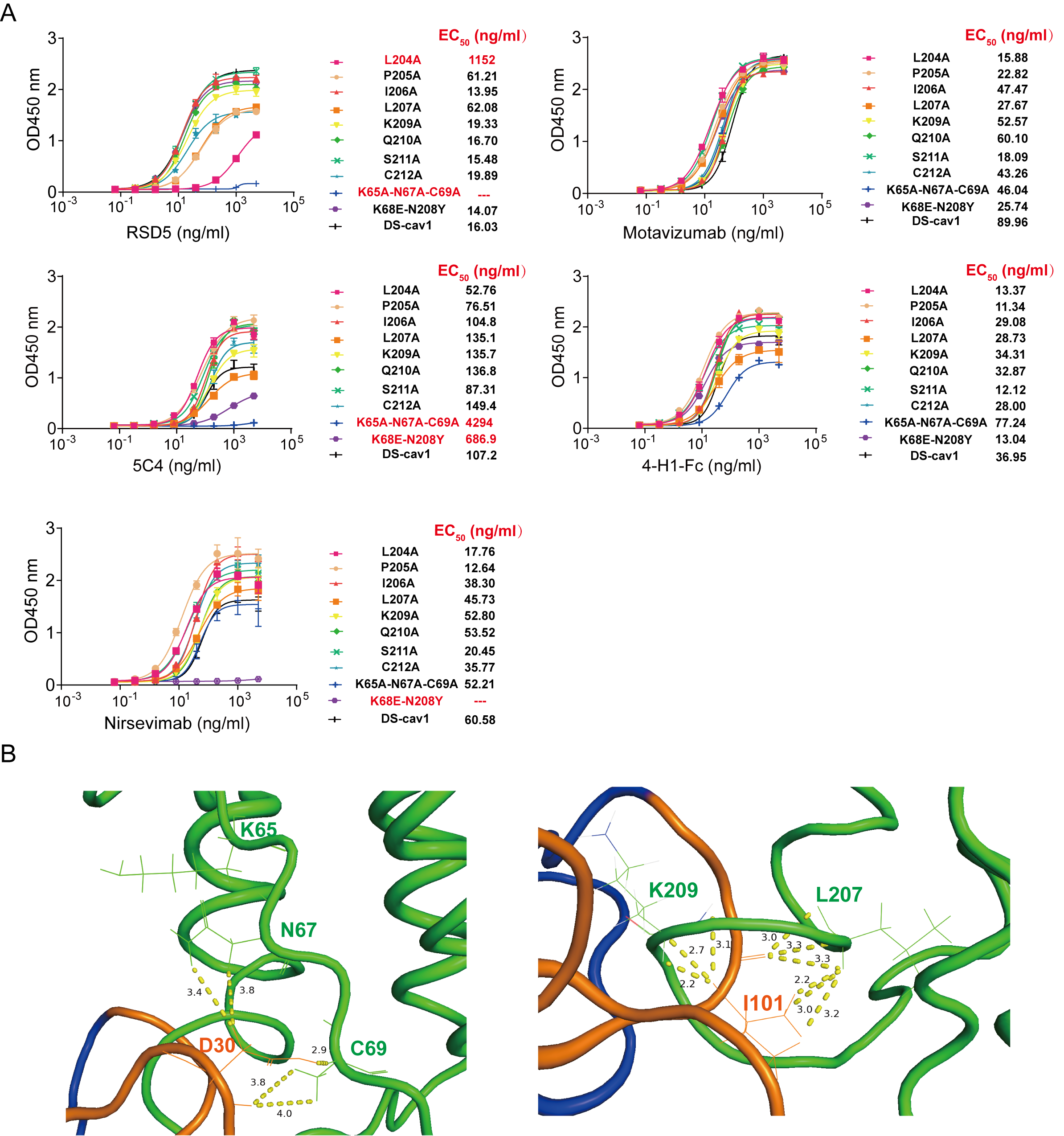

Supplement: Figure S2 — 4-H1 epitope identification. [file jvi.01285-25-s0002.tif]

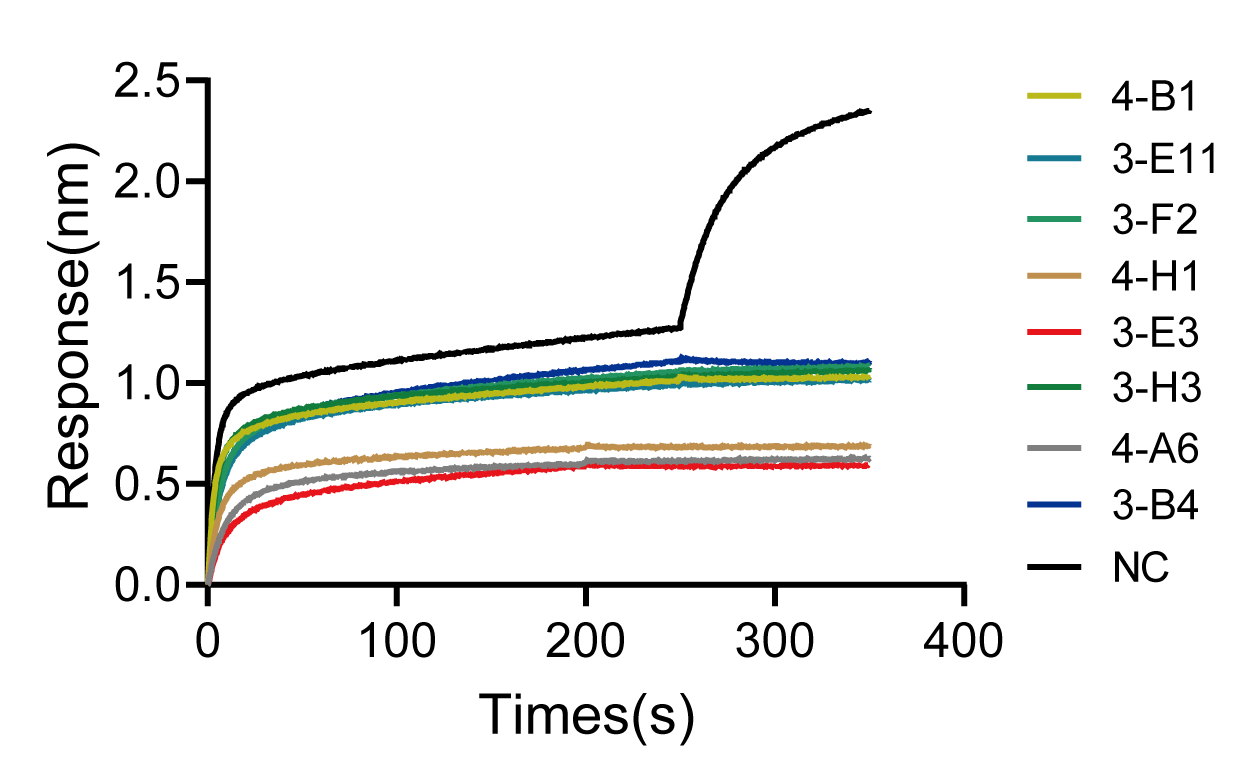

Supplement: Figure S3 — Assessment of anti-HSA nanobody binding and cross-reactivity. [file jvi.01285-25-s0003.tif]

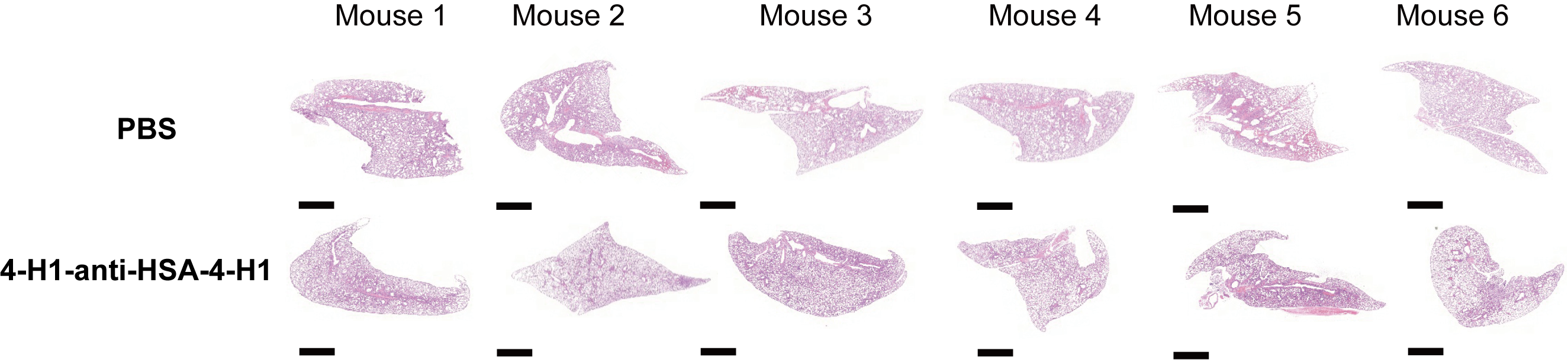

Supplement: Figure S4 — H&E staining of lung tissue sections from mice treated with PBS or 4-H1-anti-HSA-4-H1. [file jvi.01285-25-s0004.tif]
