# Supplementary material for: A novel bispecific nanobody protects mice against RSV infection via intranasal administration
Source: J Virol. 2025 Nov 24;99(12):e01285-25. doi: 10.1128/jvi.01285-25 (PMC12724135; doi:10.1128/jvi.01285-25)
Supplement: Supplemental material — Table S1; legends for Fig. S1 to S4. [file jvi.01285-25-s0005.docx]

**Fig S1. Epitope mapping via BLI-based competition assays.**

The RSV A2 DS-cav1 protein was immobilized on Ni-NTA biosensors. The biosensors were exposed to analyte antibodies (eight Fc-fused nanobodies and a non-neutralizing epitope negative control mAb, designated as NC) followed by competitor mAb D25.

**Fig S2. 4-H1 epitope identification.**

(A) ELISA curves showing binding of RSV A2 wild-type and mutated DS-cav1 to RSD5, motavizumab, 5C4, 4-H1-Fc, and nirsevimab. The EC_50_ values for the binding of each mutated DS-cav1 are listed on the right side of the panel. Residues causing a greater than 5-fold increase in EC₅₀, along with their corresponding values, were highlighted in red. The coated antigen concentration was 2 µg/ml. “---” indicates no binding. (B) Close-up view showing distance measurements between 4-H1 residues (D30 and I101) and DS-cav1 residues (K65, N67, C69, L207, K209).

**Fig S3. Assessment of anti-HSA nanobody binding and cross-reactivity.**

(A) SPR sensorgram displaying the binding of HSA to immobilized anti-HSA. HSA protein was injected at 2-fold serial dilutions ranging from 25 nM to 0.78 nM. (B) ELISA detection of the cross-binding activity of nanobodies with MSA. ELISA curves showing the binding of anti-HSA (B) and 4-H1-anti-HSA-4-H1 (C) to HSA and MSA proteins.

**Fig S4.  H&E staining of lung tissue sections from mice treated with PBS or 4-H1-anti-HSA-4-H1.**

Sections from six mice per group are shown.

**Supplementary Sequence of DS-Cav1**

MELLILKANAITTILTAVTFCFASGQNITEEFYQSTCSAVSKGYLSALRTGWYTSVITIELSNIKENKCNGTDAKVKLIKQELDKYKNAVTELQLLMQSTPATNNRARRELPRFMNYTLNNAKKTNVTLSKKRKRRFLGFLLGVGSAIASGVAVCKVLHLEGEVNKIKSALLSTNKAVVSLSNGVSVLTFKVLDLKNYIDKQLLPILNKQSCSISNIETVIEFQQKNNRLLEITREFSVNAGVTTPVSTYMLTNSELLSLINDMPITNDQKKLMSNNVQIVRQQSYSIMCIIKEEVLAYVVQLPLYGVIDTPCWKLHTSPLCTTNTKEGSNICLTRTDRGWYCDNAGSVSFFPQAETCKVQSNRVFCDTMNSLTLPSEVNLCNVDIFNPKYDCKIMTSKTDVSSSVITSLGAIVSCYGKTKCTASNKNRGIIKTFSNGCDYVSNKGVDTVSVGNTLYYVNKQEGKSLYVKGEPIINFYDPLVFPSDEFDASISQVNEKINQSLAFIRKSDELLGSGYIPEAPRDGQAYVRKDGEW

VLLSTFLGSG HHHHHH

**Table S1. Summary of nanobodies target for RSV F**

| **Nanobody** | **RSV A**  **(IC_50_, nM)** | **RSV B**  **(IC_50_, nM)** | **Epitope** |
| --- | --- | --- | --- |
| **ALX-0171** | **0.10** | **0.40** | **II (1)** |
| **F-VHHb** | **0.056** |  | **IV-VI (2, 3)** |
| **F-VHH-Cl184** | **0.4** | **21.6** | **I, III and IV (4)** |
| **F-VHH-4 /**  **F-VHH-L66** | **0.038 / 0.089** | **0.022 / 0.032** | **II, III, V and IV (4, 5)** |
| **m17 / m35** | **44.67 / 46.67** | **66.67 / 56.00** | **VI (6)** |
| **4-H1-anti-HSA-4-H1** | **0.014** | **0.0086** | **Ø** |

The IC_50_ values are all traceable to the literature, except that those of m17 and m35 were provided in μg/mL and were converted to nM based on a molecular weight of 15 kDa. The molecular weight of 4-H1-anti-HSA-4-H1 is 41.43 kDa.

**References**

1. Detalle L, Stohr T, Palomo C, Piedra PA, Gilbert BE, Mas V, Millar A, Power UF, Stortelers C, Allosery K, Melero JA, Depla E. 2016. Generation and Characterization of ALX-0171, a Potent Novel Therapeutic Nanobody for the Treatment of Respiratory Syncytial Virus Infection. Antimicrob Agents Chemother 60:6-13.

2. Schepens B, Ibañez LI, De Baets S, Hultberg A, Bogaert P, De Bleser P, Vervalle F, Verrips T, Melero J, Vandevelde W, Vanlandschoot P, Saelens X. 2011. Nanobodies® Specific for Respiratory Syncytial Virus Fusion Protein Protect Against Infection by Inhibition of Fusion. The Journal of Infectious Diseases 204:1692-1701.

3. Hultberg A, Temperton NJ, Rosseels V, Koenders M, Gonzalez-Pajuelo M, Schepens B, Ibañez LI, Vanlandschoot P, Schillemans J, Saunders M, Weiss RA, Saelens X, Melero JA, Verrips CT, Van Gucht S, de Haard HJ. 2011. Llama-derived single domain antibodies to build multivalent, superpotent and broadened neutralizing anti-viral molecules. PLoS One 6:e17665.

4. Rossey I, Hsieh CL, Sedeyn K, Ballegeer M, Schepens B, McLellan JS, Saelens X. 2021. A vulnerable, membrane-proximal site in human respiratory syncytial virus F revealed by a prefusion-specific single-domain antibody. J Virol 95.

5. Rossey I, Gilman MS, Kabeche SC, Sedeyn K, Wrapp D, Kanekiyo M, Chen M, Mas V, Spitaels J, Melero JA, Graham BS, Schepens B, McLellan JS, Saelens X. 2017. Potent single-domain antibodies that arrest respiratory syncytial virus fusion protein in its prefusion state. Nat Commun 8:14158.

6. Xun G, Song X, Hu J, Zhang H, Liu L, Zhang Z, Gong R. 2021. Potent Human Single-Domain Antibodies Specific for a Novel Prefusion Epitope of Respiratory Syncytial Virus F Glycoprotein. J Virol 95:e0048521.
